# Macrophage-Mediated Bone Formation in Scaffolds Modified With MSC-Derived Extracellular Matrix Is Dependent on the Migration Inhibitory Factor Signaling Pathway

**DOI:** 10.3389/fcell.2021.714011

**Published:** 2021-09-21

**Authors:** Moyuan Deng, Jiulin Tan, Qijie Dai, Fei Luo, Jianzhong Xu

**Affiliations:** Department of Orthopaedics, Southwest Hospital, Army Medical University, Chongqing, China

**Keywords:** mesenchymal stem cells, macrophages, extracellular matrix, osteogenic differentiation, migration inhibitory factor, osteoimmunological microenviroment

## Abstract

The positive role of macrophages in the osteogenesis of mesenchymal stem cells (MSCs) has been a recent research focus. On the other hand, MSCs could carefully regulate the paracrine molecules derived from macrophages. Human umbilical cord mesenchymal stem cells (hucMSCs) can reduce the secretion of inflammatory factors from macrophages to improve injury healing. hucMSC-derived extracellular matrix (hucMSC-ECM) has the similar effect to hucMSCs, which could combat the inflammatory response of macrophages. Additionally, MSC-derived extracellular matrix also enhanced bone regeneration by inhibiting osteoclastic differentiation of monocyte/macrophage lineage. However, whether hucMSC-ECM could improve bone formation by guiding macrophage-induced osteogenic differentiation of MSCs is unknown. Here, we present decalcified bone scaffolds modified by hucMSC-derived extracellular matrix (*DBM-ECM*), which maintained multiple soluble cytokines from hucMSCs, including macrophage migration inhibitory factor (MIF). Compared with DBM, the *DBM-ECM* scaffolds induced bone formation in an improved heterotopic ossification model of severe combined immunodeficiency (SCID) mice in a macrophage-dependent manner. Macrophages cocultured with *DBM-ECM* expressed four osteoinductive cytokines (BMP2, FGF2, TGFβ3 and OSM), which were screened out by RNA sequencing and measured by qPCR and western blot. The conditioned medium from macrophages cocultured with *DBM-ECM* improved the osteogenic differentiation of hBMSCs. Furthermore, *DBM-ECM* activated CD74/CD44 (the typical MIF receptors) signal transduction in macrophages, including phosphorylation of P38 and dephosphorylation of c-jun. On the other side, the inhibitory effects of the *DBM-ECM* scaffolds with a deficient of MIF on osteogenesis *in vitro* and *in vivo* revealed that macrophage-mediated osteogenesis depended on MIF/CD74 signal transduction. The results of this study indicate that the coordinated crosstalk of macrophages and MSCs plays a key role on bone regeneration, with an emphasis on hucMSC-ECM constructing a macrophage-derived osteoinductive microenvironment.

## Introduction

Large bone defects healing requires implants to achieve the classic stages of endochondral ossification and bone tissue regeneration, which caused by trauma, spinal injury, osteitis and tumor resection. In this mode of bone regeneration, the repair is initiated by the infiltration of immune cells, particularly macrophages. In addition to phagocytosing necrotic cells and tissue debris, macrophages initiate the recruitment of mesenchymal stem cells (MSCs) and other progenitor cells (Pajarinen et al., 2019). MSCs and other progenitor cells differentiate to form granulation tissue and ultimately form cartilage calli for bone regeneration (Pajarinen et al., 2019). The differentiation of MSCs and the subsequent formation of bone are regulated by the microenvironment. Recently, in addition to recruiting MSCs, it has been determined that macrophages play a key role in the osteogenic differentiation of MSCs and the maintenance bone tissue homeostasis (Niu et al., 2017). However, how macrophages improve bone formation remains unclear. And how to construct a macrophage-induced osteogenic microenvironment for MSCs remains unknown.

Mesenchymal stem cells (MSCs) have a good immunomodulatory ability, especially for macrophages (Swartzlander et al., 2015). MSCs derived from umbilical cord Wharton’s jelly (hucMSCs) are regarded to be significantly superior to those derived from bone marrow and adipose tissue due to their non-invasive tissue sources and, most importantly, increased immunomodulatory properties (Zhao et al., 2010; Wang et al., 2015). As MSC-derived extracellular matrix has the similar nature to native cells, the extracellular matrix of hucMSCs (hucMSC-ECM) also achieves immunomodulatory potency in combating macrophage-derived inflammation (Deng et al., 2020). Additionally, extracellular matrix or vesicles derived from MSCs inhibited the osteoclastic differentiation of monocyte/macrophage and this demonstrated that MSC-derived ECM or vesicles could improve bone formation by inhibiting osteoclastogenesis (Li et al., 2018; Hu et al., 2020). On the other hand, whether hucMSC-ECM could improve bone formation by conducting macrophage-induced osteogenic differentiation of MSCs needs to be investigated. Recent studies showed that macrophages or monocytes in a co-culture with MSCs could increase bone formation, which was mediated by Oncostain M(OSM) secreted by monocytes (Pajarinen et al., 2019). Based on hucMSC-ECM has a similar effect to hucMSCs, it is logical to hypothesize that hucMSC-ECM might induce some osteoinductive cytokines expressed by macrophages, and these cytokines improved osteogenesis of MSCs and bone formation.

**FIGURE 1:**
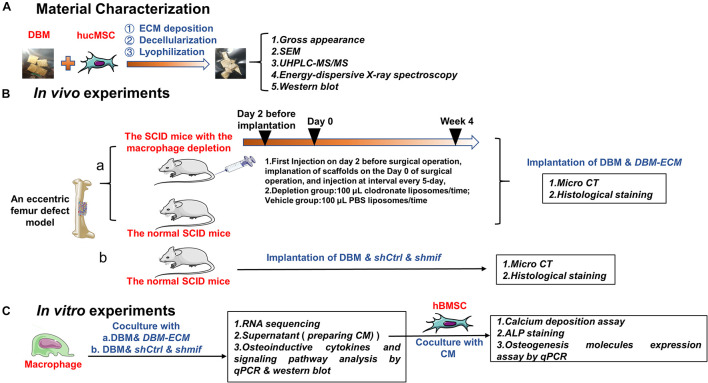
Schematic diagram of the experiments assessing macrophage-mediated bone formation of *DBM-ECM in vivo* and *in vitro*. **(A)** Material preparation and characterization. **(B)** *In vivo* experiments assessing the role of macrophages in ECM-induced bone formation **(a)** and the effect of the MIF signaling pathway on macrophage-mediated bone formation **(b)**. **(C)** *In vitro* experiments investigating the role of conditioned media of macrophages on osteogenesis of hBMSCs and the mechanism of macrophage-mediated osteogenesis of MSCs induced by ECM.

The aim of this study was to investigate whether bone regeneration of hucMSC-ECM is mediated by macrophages. Firstly, the characterization of scaffolds modified by hucMSC-derived ECM was examined. Next, the role and mechanism of hucMSC-ECM on expression of macrophage-derived paracrine osteogenic molecules were estimated. Lastly, the efficacy of hucMSC-ECM on macrophage-mediated osteogenic differentiation of bone marrow MSCs (BMSCs) and bone regeneration in improved heterotopic ossification model of severe combined immunodeficiency (SCID) mice were investigated (Figure 1).

## Materials and Methods

### Cell Culture

hucMSCs and hBMSCs were purchased from Cyagen (Cyagen Biosciences Inc., Guangzhou, China). MSCs were cultured in DMEM/F12 (HyClone, GE Healthcare Life Sciences, United States) with 10% fetal bovine serum (FBS; HyClone, United States) and 1% penicillin-streptomycin (HyClone). Passage 3-5 hucMSCs were used for the preparation of scaffolds. Passage 3-6 hBMSCs were used for osteogenic differentiation. The human monocytic cell line THP-1 (Shanghai Cell Bank, Chinese Academy of Sciences, China) was maintained in RPMI 1640 medium (HyClone, United States) supplemented with 10% FBS as previously described (Shiratori et al., 2017). THP-1 cells (5 × 10^5^/mL) were differentiated into macrophages with 50 ng/mL PMA (Sigma, United States) for 48 h and cultured in RPMI 1640 medium supplemented with 10% FBS without PMA. Cells were cultured at 37°C with 5% CO_2_.

### Preparation of Scaffolds

Demineralized bone matrix (DBM) (3 mm × 3 mm × 3 mm) was prepared with OX cancellous bone (Chongqing Datsing Bio-Tech Co., Ltd, Chongqing, China). DBM scaffolds were seeded with hucMSCs and cultured in complete medium for 12 days, rinsed with PBS, flash frozen in liquid nitrogen for 10 min, thawed in a 37°C water bath with shaking at 60 rpm for 20 min, rinsed with PBS, frozen at −80°C overnight, and freeze-dried to prepare the *DBM-ECM*. To assess the effect of MIF (macrophage migration inhibitory factor) in the *DBM-ECM* scaffolds on macrophage-mediated bone formation, *DBM-ECM*, *shCtr*l and *shmif* were prepared. The *DBM-ECM* with a deficient of MIF expression was used. Lentiviruses carrying short hairpin RNA (shRNA) were transfected into hucMSCs. hucMSCs were cultured in DMEM/F12 medium supplemented with mif shRNA or non-silencing shRNA (OBIO Technology Co., Ltd., Shanghai, China) for 24 h, and then the culture medium was changed to complete culture medium supplemented with 2.5 pM puromycin amino nucleoside (Sigma, Saint Louis, MO, United States) to eliminate untransfected cells. The cells transfected with mif (*shmif*) or the control (*shCtrl*) shRNA were used to prepare the *DBM-ECM*.

### Scanning Electron Microscopy (SEM) Analysis

Scaffolds were fixed with glutaraldehyde, dehydrated in a gradient ethanol, and sealed with tert-butyl alcohol. The scaffolds were then sprayed with gold and examined using a scanning electron microscope (Crossbeam 340; ZEISS, German) with an accelerating voltage of 1 kV and a working distance of 4–6 mm.

### Elemental Distribution

Scaffolds were determined using energy-dispersive X-ray spectroscopy at an electron acceleration voltage of 5 kV (Deng et al., 2020).

### UHPLC-MS/MS

The total protein of scaffolds (DBM and *DBM-ECM*) was extracted with 250 μL RIPA lysis buffer (Beyotime, Jiangsu, China). Protein samples were digested and assessed by LC-MS/MS (LTQ Orbitrap Velos Pro, Thermo Fisher), and data analysis was performed as previously described (Yin et al., 2014).

### Animal Model and Macrophage Depletion

All animal care and experimental protocols complied with the Animal Management Rule of the Ministry of Public Health of China (documentation 55, 2001). Ten-week-old SCID mice weighing approximately 20 ± 2 g from the Animal Experiment Center of Southwest Hospital of China were used to prepare the unilateral femoral eccentric femoral defect model. To analyze the role of macrophages in scaffold-induced bone formation, SCID mice were divided into the following two sets: those that received selective macrophage depletion and those that did not receive macrophage depletion. The method of using clodronate liposomes to deplete macrophages is well established (Cho et al., 2014; Wu et al., 2014). Forty-eight hours before surgery, depletion of the macrophages was induced by intravenous injection of 100 μL clodronate liposomes (5 mg/mL) into the tail vein (Schlundt et al., 2018). Clodronate liposome injection was repeated every 5 days to target the different phases of the bone healing process. The surgical procedure was performed as previously described (Luo et al., 2016). Mice were randomly assigned to the following two groups: the DBM group (*n* = 6 for normal mice and *n* = 6 for macrophage-depleted mice) and the *DBM-ECM* group (*n* = 6 for normal mice and *n* = 6 for macrophage-depleted mice). To estimate the role of MIF in the macrophage-mediated bone formation ability of hucMSC-ECM, mice were also randomly assigned to the following three groups: the DBM group (*n* = 6), the *shCtrl* group (*n* = 6) and the *shmif* group (*n* = 6). Bone formation was assessed by micro-CT detection and Masson’s staining at harvesting at postoperative week 4.

### Micro CT

New bone formation at week 4 was evaluated with micro-CT (Skyscan 1272, Antwerp, Belgium). The retrieved femora with muscles removed were fixed in 4% paraformaldehyde and scanned as previously described (Deng et al., 2020). The data were subsequently analyzed and imaged using CT Analyser software (version 1.16.1.0, Skyscan1272, Bruker Microct, Kontich, Belgium). 3-D pictures were created with CTvox software (version 3.2.0r1294, Skyscan1272, Bruker Microct). Relative bone volume per tissue volume (BV/TV), trabecular number (Tb. N), and bone mineral density (BMD) (0.25 g/cm^3^ CaHA of BMD phantom) were calculated for each scaffold using CTvox software (version 3.2.0r1294, Skyscan, Antwerp, Kontich, Belgium).

### Histochemistry Assessment

The scaffolds were retrieved 4 weeks postoperatively. The muscle and soft tissue were removed. Next, the scaffolds and regenerated tissue were fixed in 4% buffered paraformaldehyde, decalcified in 4% EDTA solution, embedded in paraffin and sectioned at 4–6 mm thickness. The slides were used for Masson’s trichrome staining.

### Osteogenesis-Related Assays *in vitro*

***Preparation of conditioned media (CM)*:** THP-1 cells (5 × 10^5^/mL) were seeded in 6-well plates and differentiated into macrophages; next, scaffolds cut into small pieces were added. After 2 days of induction, the medium derived from the DBM and *DBM-ECM* groups was harvested and centrifuged at 2,000 × *g* for 10 min to collect the supernatant. The conditioned medium was the DMEM/F12 mixed with the collected supernatant at a ratio of 1:1 containing 10% FBS,100 nM dexamethasone, 50 nM ascorbic acid, and 10 nM β-sodium glycerophosphate (Solarbio, Beijing, China), which was stored at −80°C for subsequent experiments (Chen et al., 2014).

***Calcium deposition assay***: To assess calcium nodule formation and calcium deposition, hBMSCs were cultured with CM for 14 days. The cells were fixed and stained with 1% Alizarin red solution (Cyagen, Guangzhou, China). For the quantitation of calcium deposition, Alizarin red binding with calcium nodules was desorbed by 10% cetylpyridinium chloride (Aladdin, Shanghai, China), and OD measurements were obtained at a wavelength of 570 nm (Li et al., 2019).

***ALP staining***: After 10 days of incubation, hBMSCs were fixed and stained with an alkaline phosphatase assay kit (Beyotime, Jiangsu, China). ALP expression was observed by microscopy (Ma et al., 2019).

### RNA Sequencing

THP-1 cells (5 × 10^5^/mL) were seeded in 6-well plates, differentiated into macrophages, and incubated with DBM and *DBM-ECM* scaffolds for 3 to 7-day. Cells were collected for RNA sequencing. Total RNA extraction, cDNA library construction, RNA sequencing and data analyses (Dif-gene-finder, Pathway analysis and GO analysis) were carried out as previously described (Novel Bio, Shanghai, China) (Deng et al., 2020). Dif-genes of interest were selected from the results of the GO term and pathway term analysis with the key gene descriptions of “osteoblast/chondrocyte differentiation, mineralization, cartilage development, bone remodeling, chondrocyte/osteoblast/cell proliferation and endochondral ossification”. Dif-genes of interest were normalized using the log 10 value and used to generate a heatmap with Heatmap illustrator (HemI.1.0, CUCKOO Work group, Hubei, China).

### Quantitative Real-Time Polymerase Chain Reaction

To estimate the profile of osteoinductive cytokines expressed by macrophages induced by scaffolds, 5 × 10^5^/mL THP-1 cells were seeded in 6-well culture plates, differentiated into macrophages, and incubated with scaffolds. After 2 days of incubation, the expression levels of macrophage genes involved in osteoinduction (TGFβ3, BMP1, BMP6, BMP2, FGF2, Wnt5a, PTHLH, OSM, WNT7β and IL-6) were examined by quantitative PCR. For the osteogenic differentiation assay, after 14 days of incubation with CM, the gene expression of hBMSCs (OPN, OCN and OSX) was examined by quantitative PCR. Briefly, total RNA was extracted from the cells with TRIzol reagent (Takara, Beijing, China) and reverse transcribed with the PrimeScript^TM^ RT reagent kit (Takara, Beijing, China) according to the manufacturer’s instructions. Real-time PCR was performed using 2 × SYBR Green PCR Master Mix. All primer sequences were obtained from Sangon Biotech Co., Ltd. (Shanghai, China), and these sequences are summarized in Supplementary Table S1.

### Western Blotting

To assess MIF deposited on scaffolds, the total protein of the DBM and *DBM-ECM* was extracted by 250 μL RIPA lysis buffer (Beyotime, Jiangsu, China). To investigate the molecular mechanism of osteoinductive cytokines expressed by macrophages induced by scaffolds, 5 × 10^5^/mL THP-1 cells were seeded in 6-well culture plates, differentiated into macrophages, and incubated with scaffolds for 0–240 min or 2 days. To estimate the knockdown level of MIF in hucMSCs, the total protein was extracted from normal cells and the cells that were transfected with lentivirus carrying mif shRNA and non-silencing shRNA. The total protein of the cells was extracted with 120 μL RIPA lysis buffer (Beyotime, Jiangsu, China), subjected to SDS-PAGE, transferred onto nitrocellulose membranes (Millipore, Billerica) and probed overnight at 4°C with specific primary antibodies against OSM, FGF2, TGFβ3, BMP2, BMP6, CD74, CD44, phspho-p38, phspho-c-jun, phspho-AKT, Erk1/2, c-jun and AKT at a dilution of 1:1000 (Abcam, United States); MIF, phspho-Erk1/2, and p38 at a dilution of 1:1000 (Cell Signaling Technology, United States); BMP1 (Bioss, Beijing, China), Wnt7β, Wnt5a, and PTHLH at a dilution of 1:500-1:1000 (Proteintech, Wuhan, China), and control antibodies Tubulin and GAPDH were used at a dilution of 1:1000 (Proteintech, Wuhan, China). Immunoreactive protein bands were visualized using ECL chemiluminescence detection plus a Western blot detection system (Viber, Viber Lourmat, France).

### Statistical Analysis

One-way ANOVA followed by a multiple comparisons test was utilized to determine the statistical significance for the micro-CT analysis (DBM, *shCtrl* and *shmif* scaffolds in normal mice), Alizarin red staining and qPCR assay (for the analysis of mRNA associated with osteogenic differentiation). T-tests were used for the analysis of the results of UHPLC-MS/MS and the qPCR assay for the analysis of osteoinductive cytokines expressed in macrophages. Two-way ANOVA followed by a multiple comparisons test was used for the micro-CT analysis of the scaffolds in normal and macrophage-depleted mice. EB-seq was used for Dif-Gene-Finder, and Fisher’s exact test was used to select significant pathways or GO terms. The results are displayed as the mean ± standard deviation. Sample numbers and repeats are for *n* ≥ 3 per group. For all the statistical tests, differences were considered to be significant if *P* < 0.05.

## Results

### MIF Abundantly Deposited on the *DBM-ECM* Scaffold Was Derived From hucMSC-Derived ECM

**FIGURE 2:**
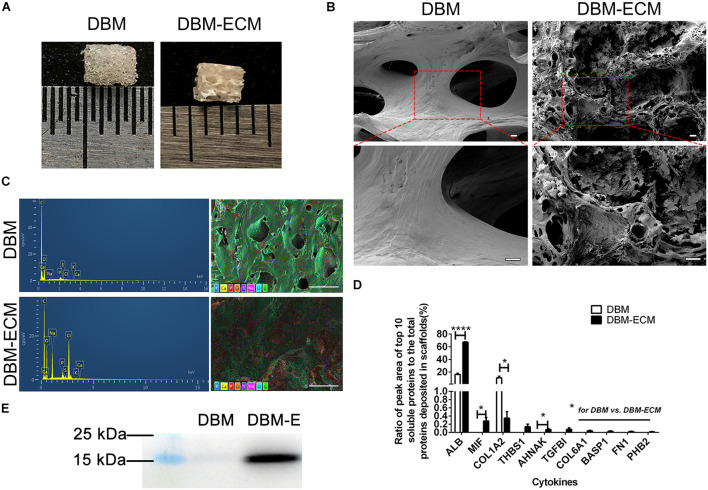
Evaluation of the surface modification of DBM scaffolds with hucMSC-ECM. **(A)** The gross appearance of the DBM scaffold with or without surface modification. **(B)** Scanning electron microscopy (SEM) images of scaffolds. **(C)** Energy dispersive spectroscopy (EDS) of scaffolds. **(D)** HPLC-MS/MS high-throughput analysis for the top 10 principal soluble protein components highly deposited in the *DBM-ECM*. **(E)** Protein expression of MIF tested by Western blot. Scale bar for the top panel of **(B)**: 100 μm, Scale bar for the down panel of **(B)**: 50 μm; Scale bar for **(C)**: 500 μm; *p < 0.05, ****p < 0.001.

In our previous studies, tissue engineering technology, followed by decellularization and lyophilization, was used to deposit hucMSC-ECM on scaffolds and enhance the osteoinductive potential of the scaffolds (Deng et al., 2016, 2020). After modification of DBM scaffolds, white, tubular hucMSC-ECM proteins appeared on the surface of the scaffolds (Figures 2A,B). The elemental distribution data showed that the contents of four elements (C,S,Cl, and Na) were remarkably different between the DBM and *DBM-ECM* scaffolds, in which the content level of C and S decreased, while the others increased (Figure 2C and Supplementary Figure S1). Furthermore, LC-MS/MS high-throughput analysis showed the presence of soluble proteins originating from the *DBM-ECM* scaffold (Figure 2D). The top 10 soluble protein peak area values were as follows: serum albumin (ALB), macrophage migration inhibitory factor (MIF), collagen alpha-1 (XII) (COL12A1), thrombospondin-1 (THBS1), transforming growth factor-beta-induced protein ig-h3 (TGFβI), neuroblast differentiation-associated protein (AHNAK), collagen alpha-1 (VI) (COL6A1), brain acid soluble protein 1 (BASP1), fibronectin (FN1) and prohibitin-2 (PHB2) (Figure 2D). Among the top ten soluble proteins, the content of macrophage migration inhibitory factor (MIF) ranked second, and a clear MIF protein band in the total protein extracted from the *DBM-ECM* was detected by Western blot (Figures 2D,E).

### Macrophage Depletion Inhibited Bone Regeneration Induced by hucMSC-ECM

**FIGURE 3:**
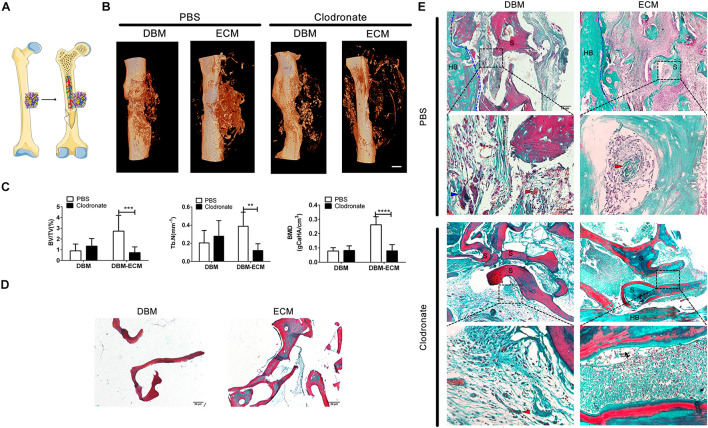
Macrophage depletion inhibited bone regeneration induced by hucMSC-ECM. **(A)** Schematic outline of surgical operation model. **(B)** 3D images of bone regeneration of the DBM and *DBM-ECM* scaffolds adjacent to the femoral shaft at 4 weeks postimplantation in SCID mice with or without macrophage depletion. **(C)** BMD, BV/TV, and Tb. N of the regenerated bone of the DBM and *DBM-ECM* scaffolds at 4 weeks postimplantation. **(D)** Masson’s staining of the DBM and *DBM-ECM* scaffolds. **(E)** Masson’s staining of the DBM and *DBM-ECM* scaffolds at 4 weeks postimplantation; HB, host bone; S, scaffold fragment; blue arrowhead, necrotic tissue; red arrowhead, new blood vessels; black arrow, granulocytes. Scale bar: 1 mm for **(B)**; Scale bar: 20 μm for **(D)**; Scale bar for upper images of panel **(E)** is 20 μm and lower images of panel **(E)** is 50 μm; **p < 0.01, ***p < 0.005, ****p < 0.001.

To estimate the role of macrophages in the bone formation ability of hucMSC-ECM, SCID mice with macrophage depletion were injected with clodronate liposomes and were compared with control mice without macrophage depletion that were injected with PBS liposomes. The DBM and the *DBM-ECM* scaffolds were harvested for micro-CT and histochemistry staining after week 4 postimplantation (Figure 3A). In control mice, the implanted DBM scaffolds formed hollow nest-like bone tissue, while the *DBM-ECM* formed dense bone tissue (Figure 3B). In the macrophage-depleted mice, all the DBM and *DBM-ECM* scaffolds exhibited similar hollow nest-like bone tissue, indicating that the surfaces of these scaffolds induced bone formation and the internal part of the scaffolds were degraded and absorbed (Figure 3B). The quantitative analysis of micro-CT data verified this observation (Figure 3C). Compared to the control mouse model, macrophage depletion did not significantly change the bone regeneration indexes of DBM scaffolds (Figure 3C). In contrast, macrophage depletion dramatically decreased the values of BV/TV, Tb. N and BMD in the *DBM-ECM* group compared with those in the control mouse model (2.73 ± 1.47- vs. 0.74 ± 0.53-fold for BV/TV, 0.38 ± 0.15- vs. 0.12 ± 0.07-fold for Tb. N, 0.26 ± 0.05 vs. 0.08 ± 0.04 folds for BV/TV; Figure 3C).

Masson’s staining of sections (5 μm) of DBM scaffolds showed red mature collagen fibers, and the collagen fibers of *DBM-ECM* scaffolds were red and green, indicating that the scaffolds had been modified by hucMSC-ECM (Figure 3D). In the control mice, Masson’s staining of implanted DBM scaffolds showed a few red collagen fibers with some necrotic tissue scattered in the scaffold pores (blue arrowhead), while the *DBM-ECM* scaffolds had been replaced by green collagen fibers indicating new bone tissue formation that was densely granular and had a rich blood vessel network (red arrowhead). On the other hand, in the macrophage-depleted mice, the collagen fiber color in the DBM and the *DBM-ECM* scaffolds was unchanged in comparison with non-transplanted scaffolds, and sparse fibrous tissue was scattered in the scaffold pores, indicating that these scaffolds had not been replaced by new bone formation. In addition, compared to the tissue in normal mice, the new tissue of the *DBM-ECM* group macrophage-depleted mice contained numerous granulocytes, indicating severe inflammation (black arrow, Figure 3E).

### hucMSC-ECM Improved Macrophage-Mediated Osteogenic Differentiation of hBMSCs

**FIGURE 4:**
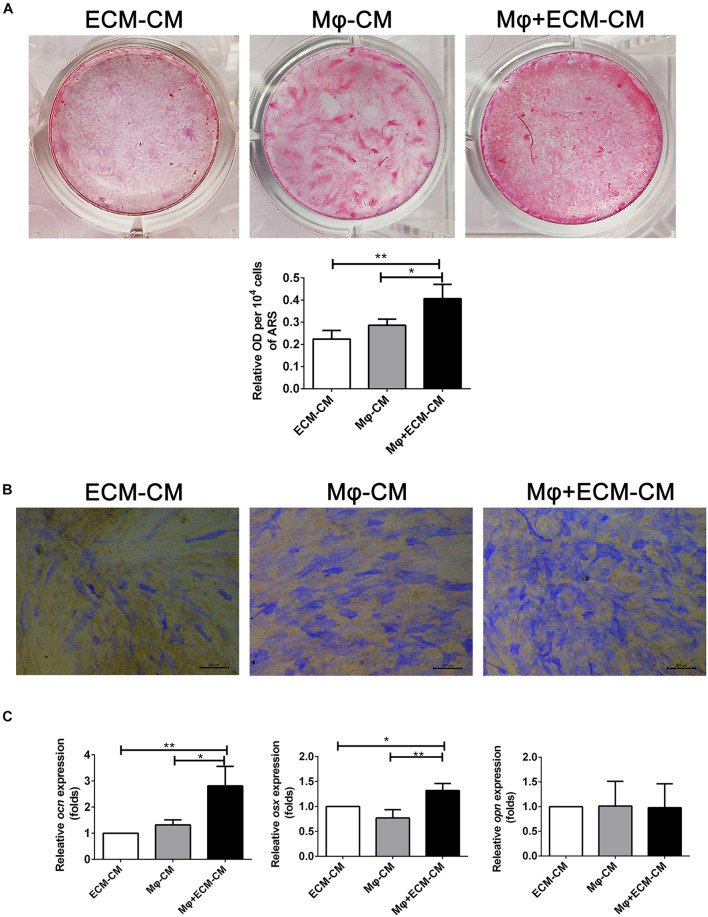
hucMSC-ECM improved macrophage-mediated osteogenesis of hBMSCs. **(A)** Alizarin red staining and OD value per 10^4^ cells of Alizarin red deposited in calcic nodules of different groups. **(B)** Alkaline phosphatase (ALP) staining of different groups. **(C)** mRNA levels of osteogenic genes of different groups; ECM-CM: conditioned medium from *DBM-ECM* scaffolds, Mφ-CM: conditioned medium from macrophages, Mφ + ECM-CM: conditioned medium from macrophages with coculture of *DBM-ECM* scaffolds. Scale bar: 200 μm for panel **(B)**. **p < 0.01; *p < 0.05.

Furthermore, the effect of macrophages on the osteogenic differentiation of MSCs induced by the *DBM-ECM* scaffolds was estimated *in vitro*. Alizarin red staining showed that conditioned medium from macrophages incubated with the *DBM-ECM* scaffolds (Mφ + ECM-CM group) induced deeper red color reaction and a higher OD value per 10 ^4^ cells compared to those in the other groups (ECM-CM and Mφ-CM), which was 82.32 ± 5.19% and 41.45 ± 11.09% higher than those of the ECM-CM and Mφ-CM groups, respectively (Figure 4A). Alkaline phosphatase (ALP) staining suggested a similar trend with the results of Alizarin red staining that the staining of the Mφ + ECM-CM group showed a deeper blue color reaction, indicating higher ALP activity (Figure 4B). Furthermore, qPCR data demonstrated that the osteocalcin (*ocn*)and osterix (*osx*) levels of the Mφ + ECM-CM group were significantly higher than those of the other two groups (2.81 ± 0.75-fold of the Mφ + ECM-CM group vs. 1.32 ± 0.19-fold of the Mφ-CM group for the *ocn* level, 1.32 ± 0.14-fold of the Mφ + ECM-CM group vs. 0.77 ± 0.17-fold of the Mφ-CM group for the *osx* level), which are key indexes for pro-osteogenic differentiation (Figure 4C). However, there were no significant differences in Osteopontin (*opn*) levels among the three groups, which is a key gene indicating bone resorption (Figure 4C; Kelly et al., 2020).

### hucMSC-ECM Enhanced the Expression of Multiple Osteoinductive Cytokines in Macrophages

**FIGURE 5:**
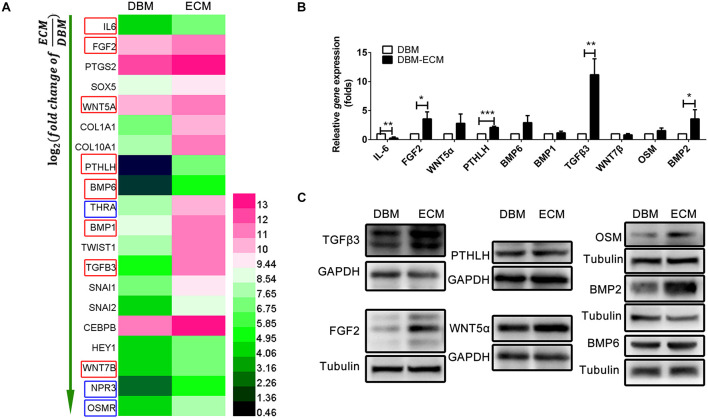
hucMSC-ECM enhanced the expression of multiple osteoinductive cytokines in macrophages. **(A)** Heatmap of upregulated Dif genes of interest in macrophages cocultured with the DBM and *DBM-ECM* scaffolds as identified by mRNA sequencing; red frame: cytokine, blue frame: receptor. **(B)** The mRNA levels of 10 cytokines in macrophages cocultured with the DBM and *DBM-ECM* scaffolds. **(C)** The protein expression of the seven cytokines in macrophages cocultured with the DBM and *DBM-ECM* scaffolds, which were screened out from qPCR results. ***p < 0.005; **p < 0.01; *p < 0.05.

To investigate the mechanism of macrophage-mediated osteogenesis induced by *DBM-ECM*, macrophages cocultured with *DBM-ECM* scaffolds were sequenced and compared with macrophages from the DBM group. The gene expression profile of macrophages cocultured with the DBM or *DBM-ECM* scaffolds was then analyzed (GSE174477)^1^. Twenty genes involved in bone formation were screened out, of which there were 8 genes that encoded for osteoinductive secretory cytokines and 3 genes that encoded for receptors (red frame for cytokine and blue frame for receptor, Figure 5A). Next, the expression of 8 screened cytokine genes and two of the other relevant genes (bone morphogenetic protein, BMP2 and Oncostatin M, OSM), a total of 10 genes, was verified by qPCR. Except for IL-6, which exhibited downregulated expression, the mRNA expression level of 4 genes in was increased in the *DBM-ECM* group compared with the expression level in the DBM group, and the difference was determined to be significant by a *t*-test (3.59 ± 1.20-fold for FGF2, 2.11 ± 0.21-fold for PTHLH, 11.20 ± 2.74-fold for TGFβ3 and 3.58 ± 1.57-fold for BMP2) (Figure 5B). To further confirm the protein expressions of cytokines screened out, except for IL-6, the expression levels of the proteins encoded for by the 9 remaining genes were verified by western blot. The results suggested that the protein expression level of TGFβ3, OSM, FGF2 and BMP2 increased, while the protein expression level of BMP6, WNT5α and PTHLH in the *DBM-ECM* group did not change compared to that in the DBM group (Figure 5C). In addition, WNT7β and BMP1 were not observed (data not shown), indicating that these proteins were only present at trace levels.

### hucMSC-ECM Upregulated the Expression of Osteoinductive Cytokines From Macrophages by the MIF Signaling Pathway

**FIGURE 6:**
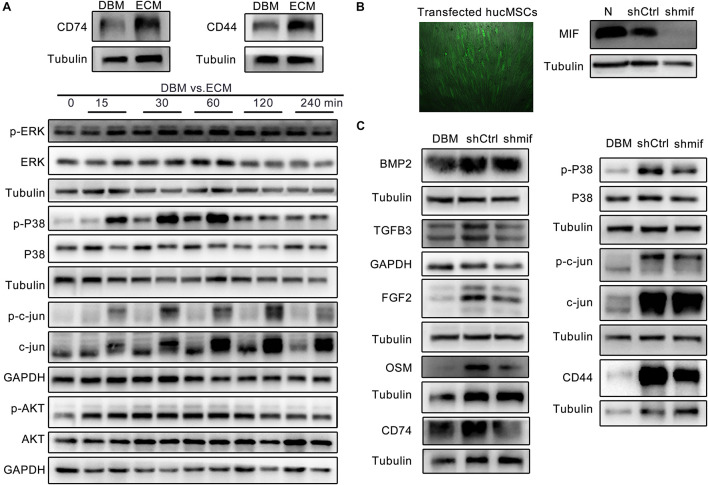
hucMSC-ECM upregulated the expression of osteoinductive cytokines in macrophages via the MIF pathway. **(A)** The protein and phosphorylation levels of key molecules involved in the MIF signaling pathway. **(B)** MIF expression of hucMSCs transfected with the lentiviral vector containing mif shRNA or no shRNA; left panel: fluorescence expression of hucMSCs transfected with the GFP lentiviral vector, right image: protein level of MIF. **(C)** The expression of cytokines and key molecules of the MIF signaling pathway derived from macrophages in the DBM, *shCtrl* and *shmif* groups.

It has been reported that MIF plays important roles in macrophage behaviors and bone healing (Liu et al., 2021; Zhu et al., 2021). Figure 2D showed that MIF deposited in hucMSC-ECM was rich in content. Thus, the role of the MIF deposited in the *DBM-ECM* scaffolds and MIF signaling pathway on the upregulation of osteoinductive cytokine expression by macrophages was estimated. CD74 and its chaperone protein CD44 have been reported to be typical MIF receptors (Jankauskas et al., 2019; Farr et al., 2020). The *DBM-ECM* scaffolds obviously enhanced the expression of CD74 and CD44, indicating that the MIF deposited in the *DBM-ECM* might activate CD74/CD44 of macrophages (Figure 6A). The latest study showed that macrophage-derived osteoinductive cytokines might be induced via CD74 downstream signaling (the MAPK and PI3K-AKT signaling pathways) (Subbannayya et al., 2016). The phosphorylation of three key transcription factors, including ERK, P38 and c-Jun of the MAPK pathway and AKT of the PI3K pathway, were tested. Western blot results showed that in the time span of 0–240 min, the *DBM-ECM* improved the phosphorylation of P38 from 15 to 60 min and the dephosphorylation of c-jun from 60 to 240 min. On the other hand, the phosphorylation levels of ERK1/2 and AKT were not affected by hucMSC-ECM (Figure 6A). To investigate the relationship between the expression of osteoinductive cytokines and MIF deposited in *DBM-ECM*, hucMSC-ECM with the knockdown of *mif* and hucMSC-ECM transfected with control shRNA were prepared. The transfection efficiency of the lentiviruses was high, as indicated by strong expression of GFP (left panel of Figure 6B). Western blot analysis showed that MIF expression in the *shCtrl* group decreased slightly compared to that in the normal hucMSCs without transfection of lentiviral vector, while MIF expression in the *shmif* group was almost completely suppressed (right panel of Figure 6B). Furthermore, after macrophages were incubated with scaffolds (DBM, the *shCtrl* and *shmif* scaffolds), the expression of the previously screened cytokines and the aforementioned signaling pathways was tested. Among the 4 cytokines (BMP2, TGFβ3, FGF2 and OSM), only the BMP2 level among the 3 groups (DBM, *shCtrl* and *shmif*) was not significantly changed, suggesting that MIF deposited in hucMSCs-ECM did not improve BMP2 expression (Figure 6C). The expression levels of TGFβ3, FGF2 and OSM in the *shmif* group were heavily decreased compared with those in the *shCtrl* group, indicating that MIF deposited in the ECM played a major role in upregulating the expression of these three cytokines (Figure 6C). In addition, the expression of CD74/CD44 and phosphorylation of P38 in the *shmif* group were inhibited compared with those in the *shCtrl* group, while dephosphorylation of c-jun was not changed as a result of MIF knockdown (Figure 6C).

### hucMSC-ECM Improved Macrophage-Mediated Osteogenesis of hBMSCs and Bone Formation by MIF Signaling Pathway

**FIGURE 7:**
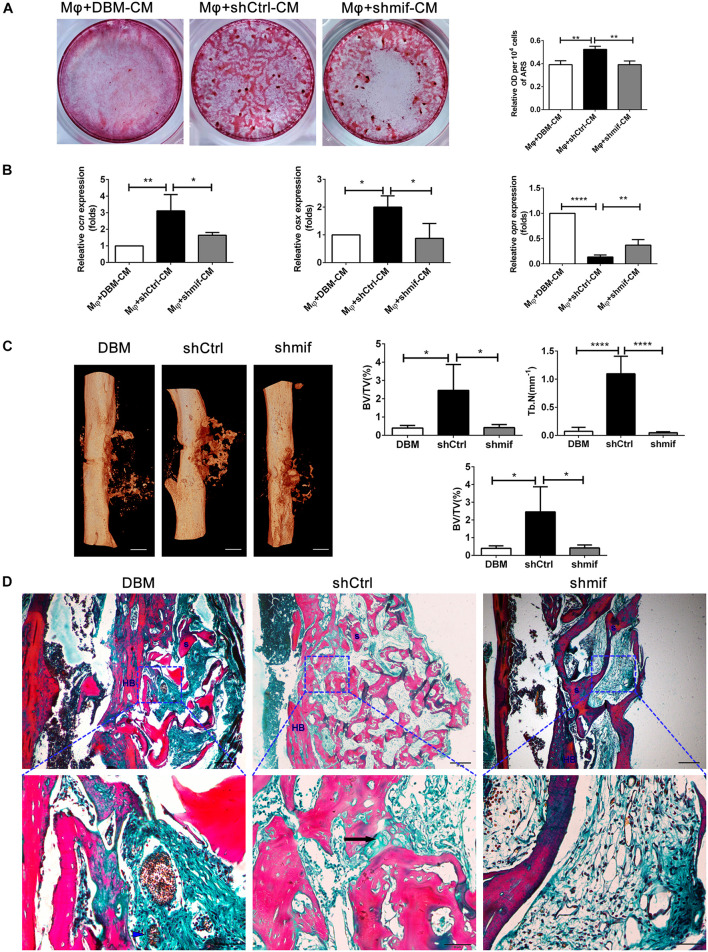
hucMSCs-ECM improved macrophage-mediated osteogenesis of hBMSCs and bone formation via the MIF signaling pathway. **(A)** Alizarin red staining and OD value per 10^4^ cells of Alizarin red deposited in calcic nodules of different groups. **(B)** mRNA levels of osteogenic genes of different groups. **(C)** Micro-CT analysis of regenerated bone induced by different scaffolds at 4 weeks postimplantation. **(D)** Masson’s staining for regenerated bone induced by different scaffolds at 4 weeks postimplantation; HB, host bone; S, scaffold fragment; blue arrowhead, necrotic tissue; black arrow, endochondral ossification. Mφ + DBM-CM: the conditioned medium from macrophages cocultured with DBM scaffolds; Mφ + shCtrl-CM: the conditioned medium from macrophages cocultured with the *shCtrl* scaffolds; Mφ + shmif-CM: the conditioned medium from macrophages cocultured with the *shmif* scaffolds. Scale bar: 1 mm for **(C)**, scale bar for upper images of panel **(D)** is 20 μm and lower images of panel **(D)** is 50 μm; ****p < 0.001; **p < 0.01; *p < 0.05.

To further investigate the role of the MIF signaling pathway in macrophage-mediated osteogenesis and bone formation induced by *DBM-ECM*, Alizarin red staining and the expression levels of genes involved in osteogenic differentiation *in vitro* and bone regeneration *in vivo* were compared between the *shCtrl* and *shmif* groups. Compared with the Mφ + DBM-CM group, Alizarin red staining showed increasingly larger calcic nodules in the Mφ + shCtrl-CM group, which had a higher OD value per 10^4^ cells (2.62 ± 0.14-fold in the Mφ + shCtrl-CM group vs. 1.96 ± 0.17-fold in the Mφ + DBM-CM group). The BMSCs in the Mφ + shmif-CM group detached during the 14-day culture period, and Alizarin red staining showed no cell or calcic nodules in the middle of the 6-well plate, and the OD value per 10^4^ cells also decreased (1.96 ± 0.16-fold) (Figure 7A). qPCR results showed that the expression of *ocn* and *osx* (indicating osteogenic differentiation) in the Mφ + shCtrl-CM group remarkably increased compared with that in the Mφ + DBM-CM group (3.39 ± 1.21-fold in the Mφ + shCtrl-CM group for *ocn*, 2.00 ± 0.40-fold in the Mφ + shCtrl-CM group for *osx*), while it was obviously decreased compared with that of the Mφ + shmif-CM group (1.69 ± 0.20-fold in the Mφ + shmif-CM group for *ocn*, 0.93 ± 0.62-fold in the Mφ + shmif-CM group for *osx*). In addition, the *opn* level of the Mφ + shCtrl-CM group showed a striking decrease compared with that of the Mφ + DBM-CM group (0.14 ± 0.04-fold of the Mφ + shCtrl-CM group), and the deficient of mif slightly reversed this trend (0.37 ± 0.11-fold of the Mφ + shmif-CM group) (Figure 7B). Furthermore, the bone formation ability of the three groups *in vivo* verified the results of qPCR and Alizarin red staining *in vitro*. 3D micro-CT images showed that more new bone tissue appeared in the *shCtrl* group and less new bone tissue appeared in the *shmif* group and DBM group. Three key evaluation indexes of micro-CT (relative bone volume, trabecular number and bone mineral density) also maintained a similar trend. The BV/TV, Tb. N and BMD of the *shmif* group (0.43 ± 0.14-fold for BV/TV, 0.05 ± 0.01-fold for Tb. N and 0.08 ± 0.02-fold for BMD) was lower than that of the *shCtrl* group (2.46 ± 1.24-fold for BV/TV, 1.10 ± 0.31-fold for Tb. N and 0.13 ± 0.03 folds for BMD) (Figure 7C). Based on Masson’s staining, the implanted DBM retained many red scaffold fragments that had not been replaced by new bone tissue and some necrotic tissue was scattered between the scaffold fragments. The *shCtrl* scaffold fragment was replaced with new bone tissue, as indicated by endochondral ossification appearing between the scaffold fragments. The *shmif* scaffolds did not show any sign of degradation or replacement by new bone tissue as indicated by the maintenance of the scaffold structure by red collagen fibers with few green fibers (Figure 7D).

## Discussion

Osteogenic differentiation and bone formation are determined by the microenvironment, which involves many cells and cytokines, including innate immune cells and the extracellular matrix niche (Torossian et al., 2017; Rowley et al., 2019). Recently, with the emergence of osteoimmunology, the increasing studies have focused on regulating macrophage behavior to create an osteoinductive niche (Takayanagi et al., 2000; Chen et al., 2015). In this study, we created a macrophage-mediated osteoinductive niche based on hucMSC-ECM. Compared to some previous ECM-based immunomodulatory biomaterials incorporated with ECM-derived molecules, the modification by the facile tissue engineering technique not only changes the elemental distribution of DBM scaffolds, but also deposits the full natural context of the original ECM protein on DBM scaffolds, containing multiple bioactive soluble cytokines (Figures 2C,D; Rowley et al., 2019). The variety of cytokines deposited on scaffolds improves the ability of macrophages to express some osteoinductive cytokines, including FGF2, TGFβ3, OSM and BMP2, which provides an osteogenic microenvironment (Figures 4–6). Indeed, approximately eight osteoinductive genes were identified by RNA sequencing and qPCR, but the protein expression of only four cytokines was increased (Figure 5). Previous studies revealed that MSCs can induce monocytes/macrophages to secrete OSM, and OSM from activated macrophages could promote osteoblastic differentiation and mineralization of human muscle-derived stromal cells surrounding neurogenic heterotopic ossification (Nicolaidou et al., 2012; Torossian et al., 2017). Our results verified that hucMSC-ECM can also upregulate the expression of OSM from macrophages and improve bone formation of the *DBM-ECM* scaffolds attached to the femur with an improved heterotopic ossification model (Figures 3, 5, 7). In addition, BMP2 levels had been reported to increase in the medium of cocultured M2 macrophages and MSCs (Ekstrom et al., 2013). This study also achieved similar results that hucMSC-ECM could also upregulate macrophage-derived BMP2 expression. Furthermore, the *DBM-ECM* scaffolds enhanced FGF2 and TGFβ3 expression in addition to BMP2 (Figure 5). This result is consistent with observation of Chen et al. in which β-tricalcium phosphate increased the mRNA levels of BMP2, VEGF and TGFβ3 in macrophages (Chen et al., 2014, 2015). The difference is that *DBM-ECM* scaffolds can’t upregulate VEGF level of macrophages. It’s worth noting that *DBM-ECM* upregulated FGF2 mRNA and protein expression levels, which has rarely been observed in previous studies. Lastly, the conditioned media derived from macrophages induced by the *DBM-ECM* (Mφ + ECM-CM group) improved osteogenic differentiation and mineralization level of hBMSCs compared to ECM-CM group and Mφ-CM group (Figure 4). Importantly, Mφ + ECM-CM upregulated the mRNA levels of OCN and OSX in hBMSCs except for OPN (Figure 4C). OCN and OSX are the key molecules indicating osteogenic differentiation, while OPN is thought to enhance bone resorption by anchoring osteoclasts to bone matrix (Fukuda et al., 2018; Si et al., 2020; Sun et al., 2020). These results suggested that *DBM-ECM in vitro* could promote osteogenesis of BMSCs in a macrophage-mediated manner.

In this study, an improved heterotopic ossification model of SCID mice with clodronate liposomes or not was used to estimate the role of macrophages on bone formation of the *DBM-ECM* scaffolds. SCID mouse has no functional T and B cells, but has functional macrophages, which is usually used to build the model with engraftment human organ or cell to avoid rejection caused by human derived proteins in normal mice (Barry et al., 1991; Ronchese and Hausmann, 1993; Andoh et al., 2003). In this study, SCID mice were suitable for DBM scaffolds modified by human umbilical cord mesenchymal stem cells derived ECM (the *DBM-ECM*). Clodronate liposomes are usually used to deplete macrophages *in vivo*, and SCID mice with clodronate liposomes were used for estimating the role of macrophages on bone formation of the *DBM-ECM* (Beatty et al., 2015; Han et al., 2019; Michalski et al., 2019). In addition, heterotopic ossification (HO) model is suitable for estimating osteoinductive effect of scaffolds *in vivo*, which consists of ectopic bone formation within soft tissues following surgery or trauma (Shimono et al., 2011). Muscle pouch model is usually as a HO model and used to estimate ectopic bone-formation of scaffolds with or without medicine (Dong et al., 2021). In this study, an improved heterotopic ossification model was a unilateral femoral eccentric femoral defect model with the destruction of cortical bone and overflow of bone marrow, which brought much more macrophages from bone marrow compared to the muscle pouch model (Figure 3A). This improved heterotopic ossification model can much better reflect the role of macrophages on bone formation.

This improved heterotopic ossification model introduced a new understanding of the mechanism of MSC-induced bone formation. The earlier hypothesis was that exogenous MSCs differentiated into osteoblasts and then formed bone tissue *in vivo* (Kuznetsov et al., 1997). With the increasing evidences that massive exogenous MSCs would be dead in the early postimplantation period, the role of exogenous MSCs is recruiting endogenous stem cells to participate in bone regeneration (Caplan and Correa, 2011; Vanden, 2014). In this study, the comparison of normal and macrophage-depleted mice revealed by micro CT images that the indexes for new bone in DBM scaffolds were not significantly different, while the indexes of the *DBM-ECM* scaffolds implanted in macrophage-depleted mice were heavily decreased, suggesting that macrophages are required for bone formation induced by hucMSC-ECM (Figure 3). As hucMSC-ECM was similar to intact cells, and it is logical to assume that bone formation enhanced by exogenous hucMSCs might depend on the regulation of macrophages, similar to hucMSC-ECM. This new understanding of this mechanism is different from previous hypotheses and emphasizes that exogenous MSCs promote bone regeneration in a macrophage-mediated manner.

The results of this study furtherly confirmed the positive role of the MIF/CD74 signaling pathway in bone formation. Previously, MIF was regarded as a pleiotropic inflammatory cytokine involved in exaggerated inflammation and immunopathology (Farr et al., 2020). Recently, it has become appreciated that the MIF signaling pathway protects against injury and promotes repair in many tissues, including the intestine, lung, neurologic system, heart, kidney, liver, skin and hair (Farr et al., 2020; Oh et al., 2020). However, the role of MIF in bone disease healing is controversial. Shin Onodera et al. demonstrated that transgenic mice overexpressing MIF exhibit osteoporosis, and some studies have also revealed that MIF induced osteoclastogenesis in arthritic mice (Onodera et al., 2006; Gu et al., 2015). Additionally, the MIF inhibitor 4-IPP reverses osteoclast formation (Zheng et al., 2019). On the other hand, MIF-deficient mice exhibited impaired fracture healing (Kobayashi et al., 2011). In addition, mice with deletion of CD74 (the receptor of MIF) exhibited enhanced osteoclastogenesis and decreased bone mass (Mun et al., 2013). The latest studies revealed that MIF plays a protective role in osteoarthritis (Liu et al., 2021). It has even been documented that MIF induces TNF production in monocytes, activates β-catenin in osteoblasts and promotes the mineralization of osteoblasts, indicating that the MIF/CD74 axis is linked to inflammation and new bone formation seen in ankylosing spondylitis (Ranganathan et al., 2017). In this study, hucMSC-ECM enhanced the expression of osteoinductive cytokines (FGF2, TGFβ3, OSM and BMP2) in macrophages and improved osteogenic differentiation of BMSCs (Figures 4, 5). MIF abundantly deposited on the *DBM-ECM* was derived from hucMSC-ECM, and this finding was consistent with that in Hyun Ah Oh et al.’s study, which showed that the content of MIF was the highest out of 57 growth factors in the conditioned medium of human umbilical cord blood-derived MSCs (Figures 2D,E; Oh et al., 2020). Furthermore, the Western blot results demonstrated that hucMSC-ECM with mif knockdown decreased the expression of osteoinductive cytokines (FGF2, TGFβ3 and OSM, not BMP2) in macrophages (Figure 6C). The downregulation of cytokine levels resulted in the decreases in calcium deposition and the mRNA expression of osteogenic genes in BMSCs (Figures 7A,B). Furthermore, the implanted *shmif* scaffolds *in vivo* achieved a small bone mass, which was significantly smaller than that of *shCtrl* scaffolds (Figure 7C). Many fragments of the *shmif* scaffolds were not replaced by new bone tissue, whereas new tissue and endochondral ossification was observed with *shCtrl* scaffolds (Figure 7D). These results revealed that MIF deposited in the *DBM-ECM* plays an important role in upregulating the osteoinductive cytokine expression level in macrophages, thus improving bone formation.

**FIGURE 8:**
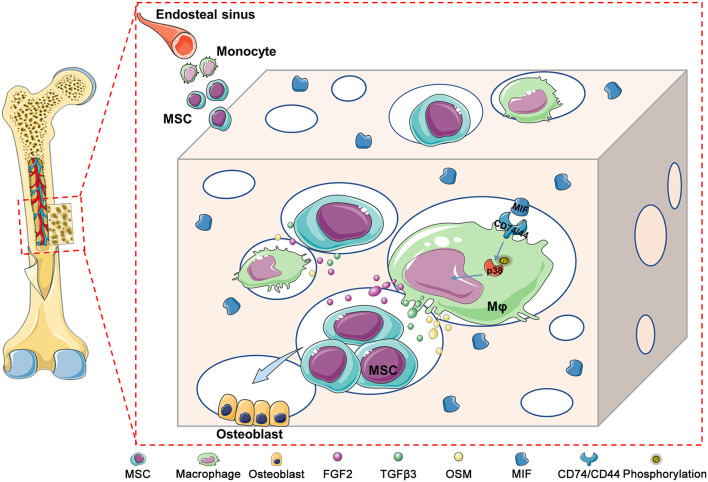
Schematic diagram illustrating the underlying mechanism of macrophage-mediated bone formation in DBM modified by hucMSC-ECM. Briefly, *DBM-ECM*-induced bone regeneration depends on the osteogenic niche constructed by macrophage-derived paracrine molecules. MIF highly deposited in the *DBM-ECM* scaffolds activates CD74/CD44 expression in macrophages, subsequently enhances the phosphorylation of P38 and finally upregulates the expression of multiple osteoinductive cytokines, including FGF2, TGFβ3 and OSM, in macrophages, which directs the osteogenesis of BMSCs and promotes bone regeneration.

In addition, CD74 is a known MIF receptor, and CD44 is a coreceptor (Farr et al., 2020). The MIF/CD74 signaling pathway induces tissue repair by triggering the activation of AMP-activated protein kinase (AMPK) and the PI3K-Akt signal transduction cascade (Subbannayya et al., 2016; Farr et al., 2020). In this study, western blot results showed that *DBM-ECM* upregulated the levels of CD74 and CD44, the phosphorylation of P38 and the dephosphorylation of c-jun, while the knockdown of *mif* downregulated the expression of CD74/CD44 and p-P38 and did not affect the MAPK-c-jun or MAPK-ERK pathway. These results suggested that MIF in the *DBM-ECM* activated the CD74/CD44-P38 signal cascade (Figures 6A,C, 8).

In conclusion, we constructed a macrophage-mediated niche that could guide the osteogenic differentiation of BMSCs and promote bone regeneration. We revealed the underlying mechanism of the MIF signaling cascade in the osteogenesis of BMSCs by an indirect immunomodulatory manner. Overall, the results of this study highlight that the osteoimmunomodulatory niche created by MSC-ECM might hold potential for bone healing.

## Data Availability Statement

The datasets presented in this study can be found in online repositories. The names of the repository/repositories and accession number(s) can be found below: https://www.ncbi.nlm.nih.gov/geo/query/acc.cgi?acc=GSE174477.

## Ethics Statement

The animal study was reviewed and approved by the Animal Management Rule of the Ministry of Public Health, China (documentation 55, 2001).

## Author Contributions

MD, FL, and JX conceived and designed the study. MD completed the experiments, including animal modeling, western blotting, SEM, collected and analyzed the data, and drafted and edited the manuscript. JT completed the experiments, including cell culture, surface modification of DBM, qPCR, and osteogenesis-related assays. QD performed micro CT analysis and results statistics. All authors contributed to the article and approved the submitted version.

## Conflict of Interest

The authors declare that the research was conducted in the absence of any commercial or financial relationships that could be construed as a potential conflict of interest.

## Publisher’s Note

All claims expressed in this article are solely those of the authors and do not necessarily represent those of their affiliated organizations, or those of the publisher, the editors and the reviewers. Any product that may be evaluated in this article, or claim that may be made by its manufacturer, is not guaranteed or endorsed by the publisher.

## Footnotes

1
https://www.ncbi.nlm.nih.gov/geo/query/acc.cgi?acc=GSE174477
