# Supplementary material for: Macrophage-Mediated Bone Formation in Scaffolds Modified With MSC-Derived Extracellular Matrix Is Dependent on the Migration Inhibitory Factor Signaling Pathway
Source: Front Cell Dev Biol. 2021 Sep 21;9:714011. doi: 10.3389/fcell.2021.714011 (PMC8490662; doi:10.3389/fcell.2021.714011)
Supplement: Supplementary file 1 [file Data_Sheet_1.pdf]

Supplemental Figure 1

DBM scaffolds

EDS images

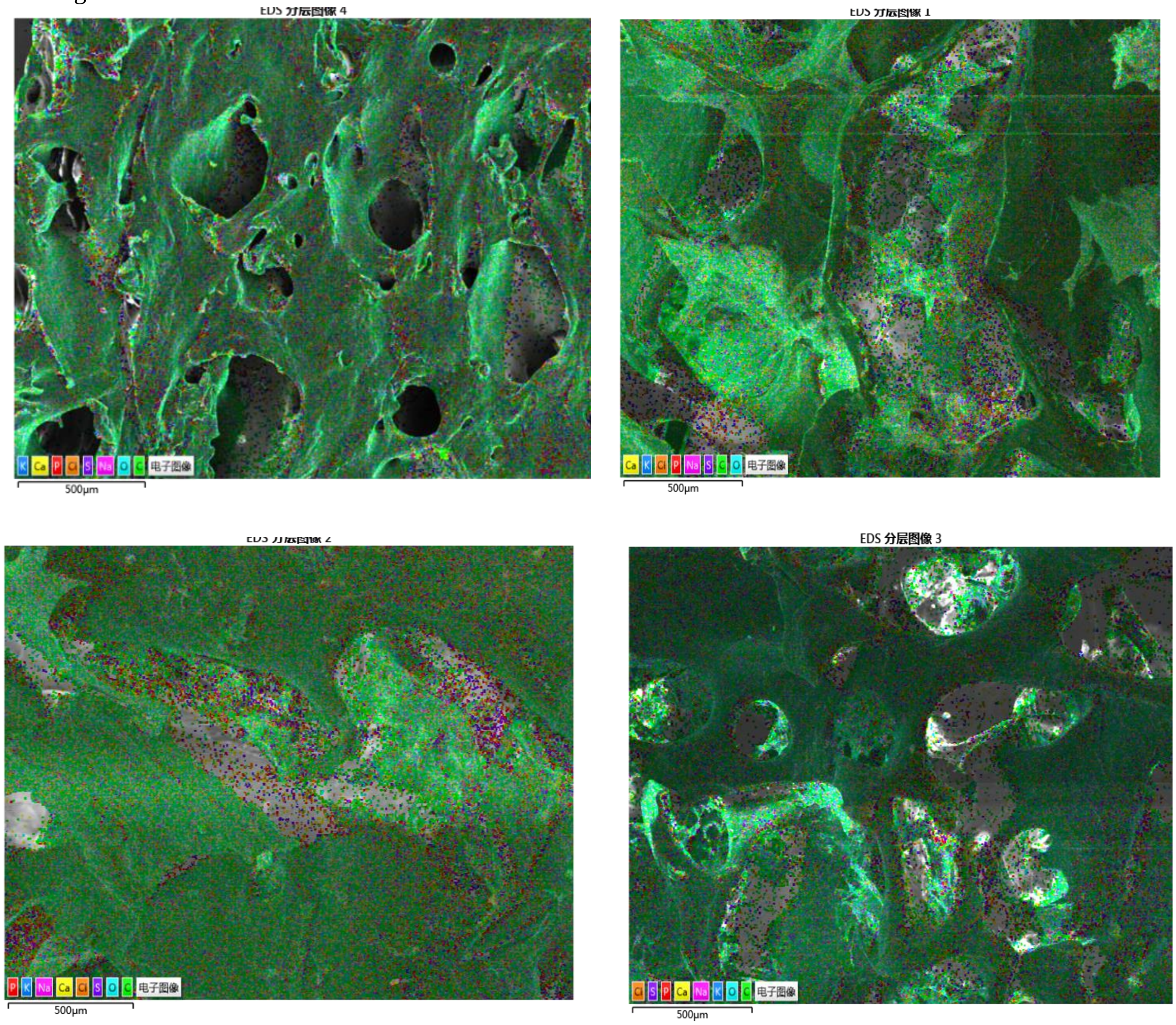

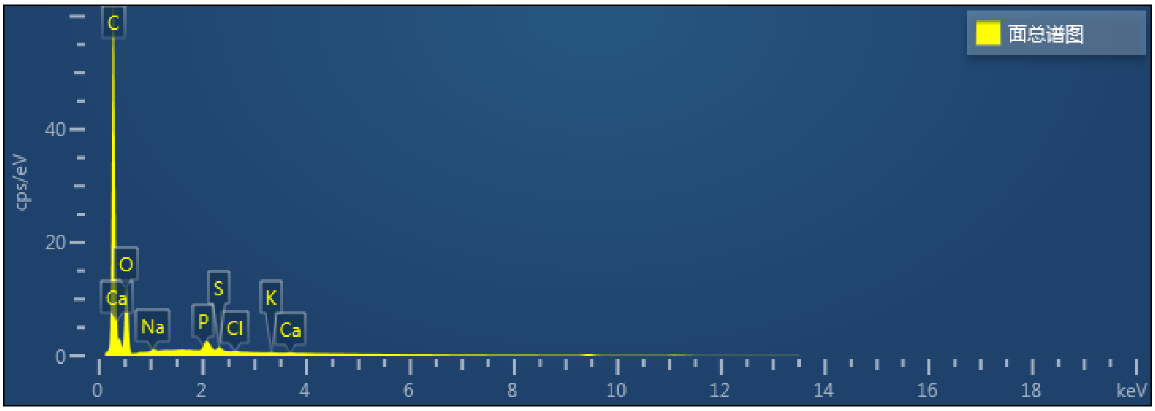

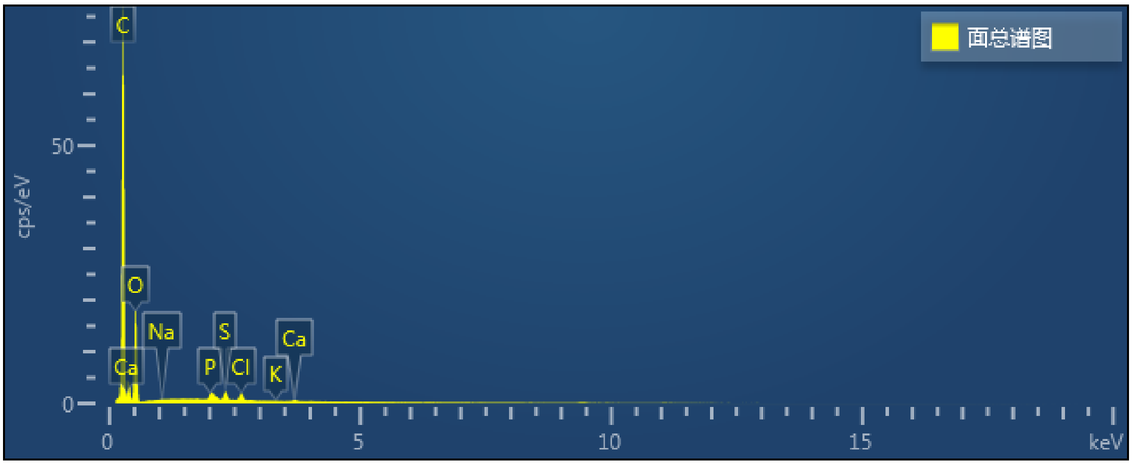
面总谱图
cps/eV
50
0
C
O
Na
S
Ca
Ca
P
Cl
K
0
5
10
15
keV

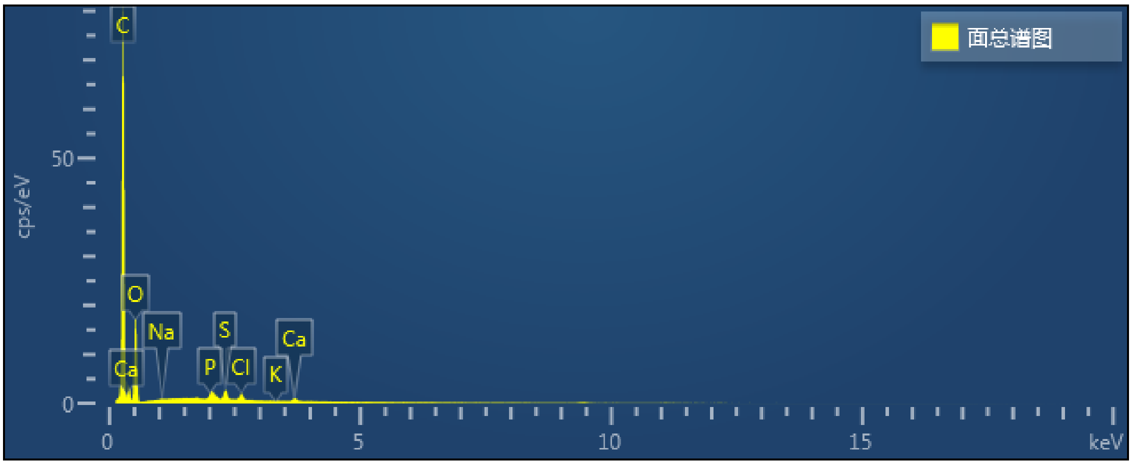
面总谱图
cps/eV
50
0
C
O
Na
S
Ca
Ca
P
Cl
K
0
5
10
15
keV

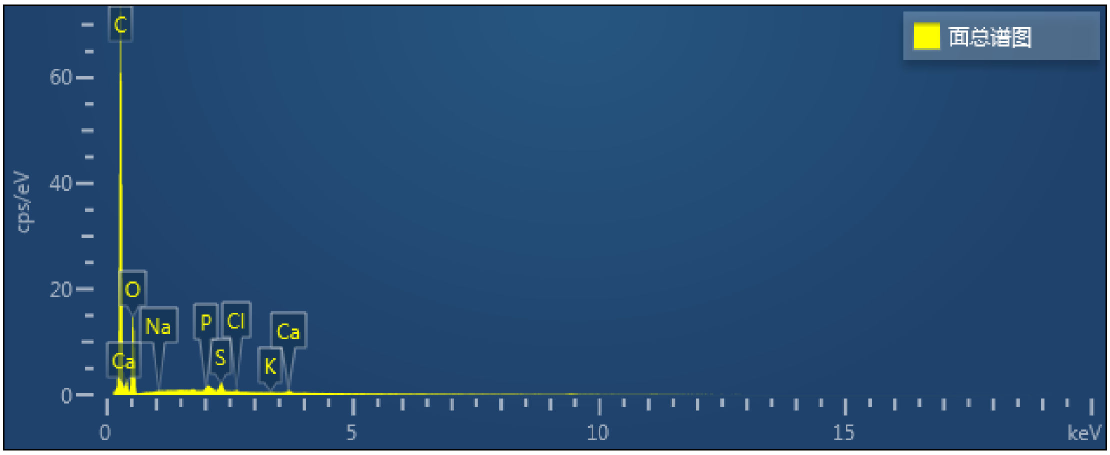
面总谱图
cps/eV
60
40
20
0
C
O
Na
P
Cl
Ca
Ca
S
K
0
5
10
15
keV

The elemental distribution of scaffolds

Element	Atomic perce of DBM scaffolds (%)	Element	Atomic perce of DBM scaffolds (%)	Element	Atomic perce of DBM scaffolds (%)	Element	Atomic perce of DBM scaffolds (%)
C	79.32	C	78.92	C	79.86	C	80.16
O	20.5	O	20.78	O	19.8	O	19.62
Na	0.07	Na	0.01	Na	0.02	Na	0.01
P	0	P	0.05	P	0.05	P	0.02
S	0.07	S	0.12	S	0.14	S	0.13
Cl	0.02	Cl	0.1	Cl	0.09	Cl	0.02
K	0.01	K	0	K	0	K	0
Ca	0.01	Ca	0.02	Ca	0.05	Ca	0.04
Total:	100	Total:	100	Total:	100	Total:	100

DBM-ECM scaffolds

EDS images

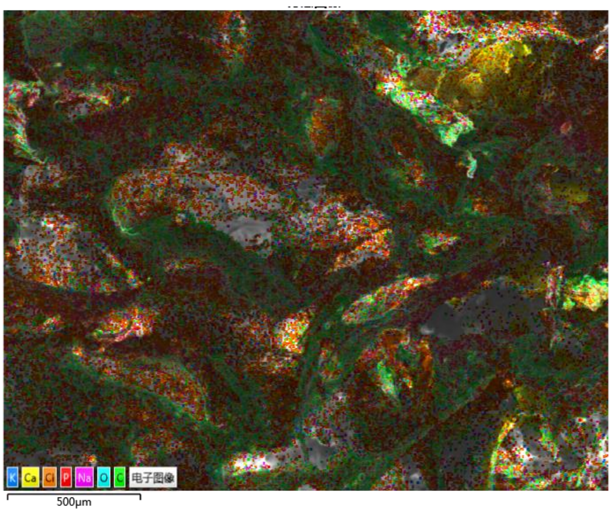

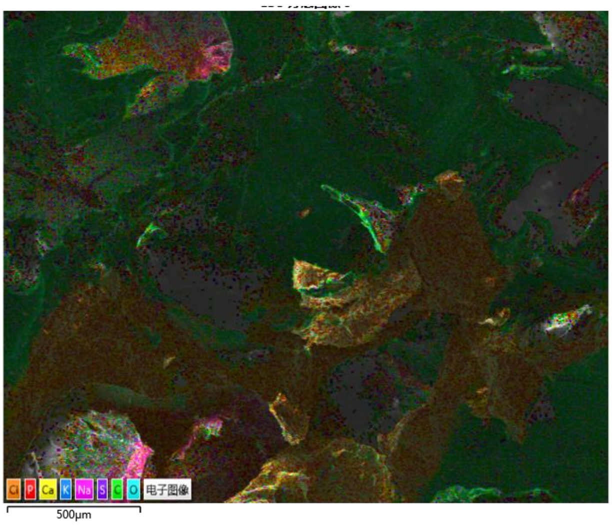

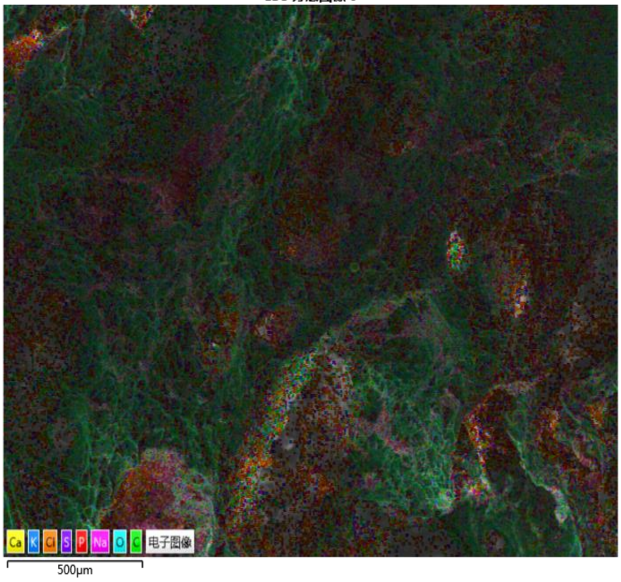
Ca K Cl S P Na O C 电子图像
500µm

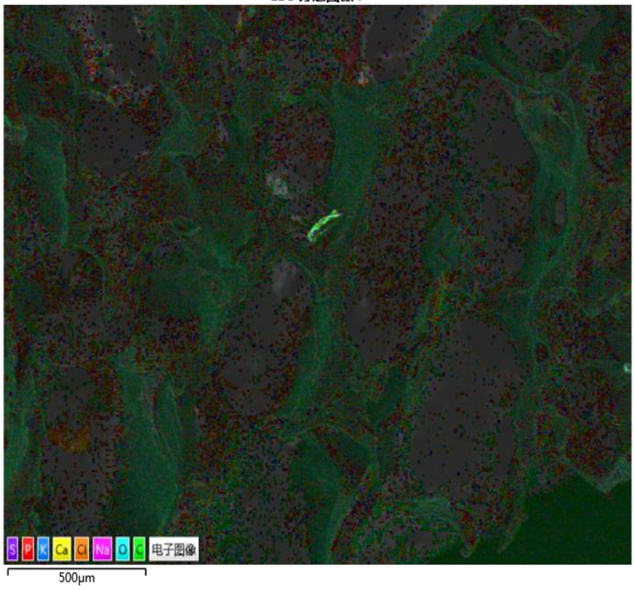
S P K Ca Cl Na O C 电子图像
500µm

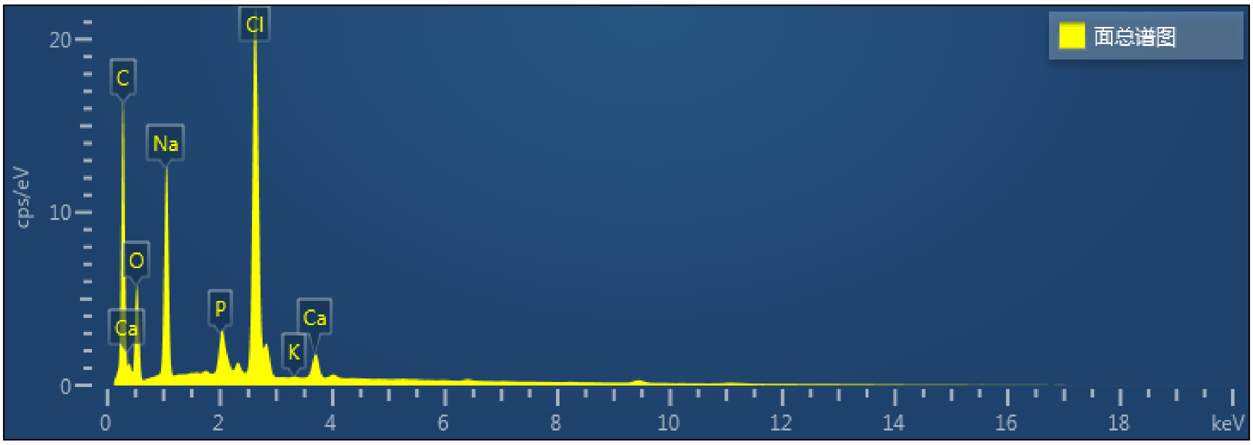
面总谱图
cps/eV
0
10
20
C
Na
O
Ca
P
Cl
K
Ca
0
2
4
6
8
10
12
14
16
18
keV

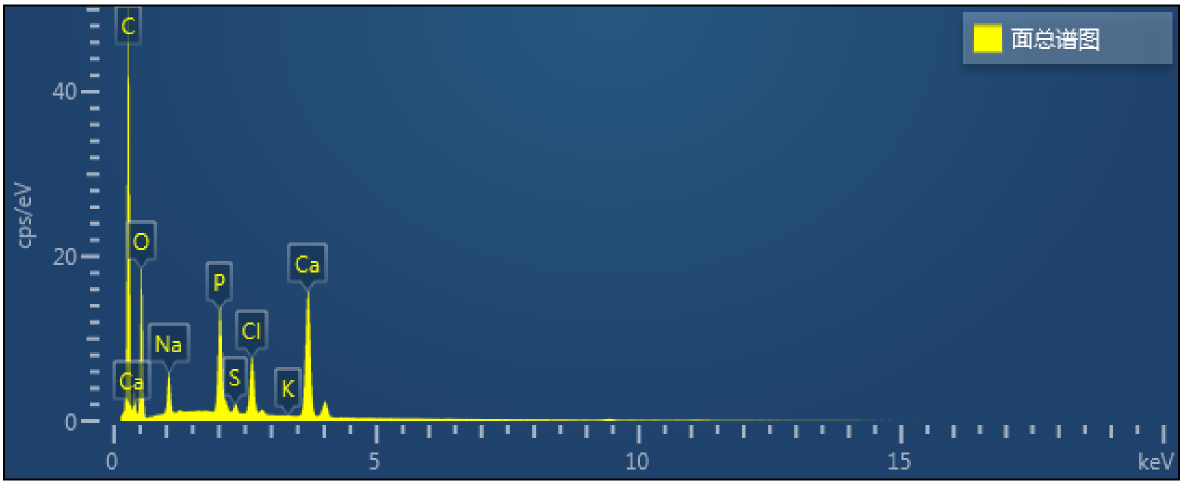
面总谱图
cps/eV
0
20
40
C
O
P
Ca
Na
Cl
Ca
S
K
0
5
10
15
keV

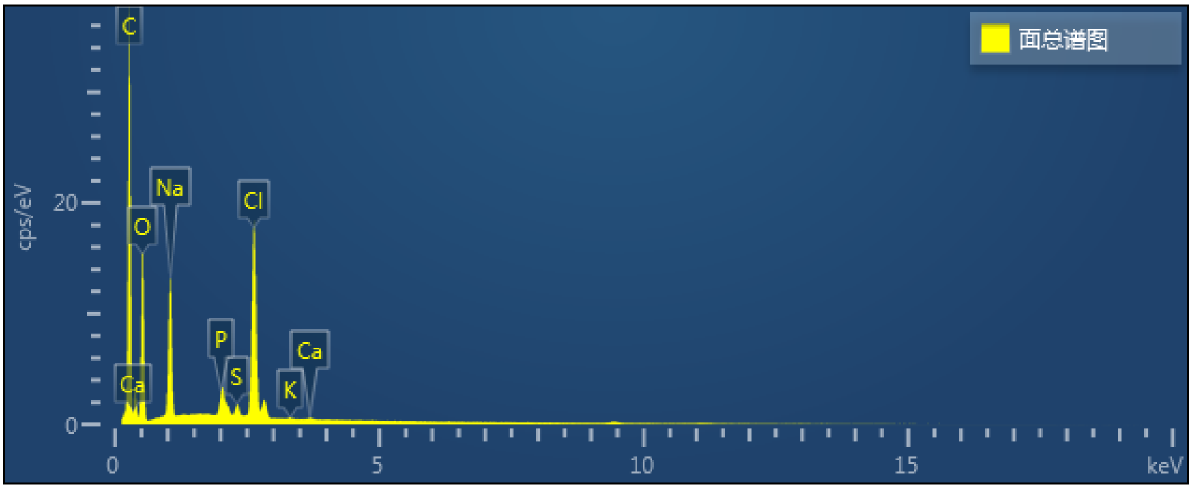

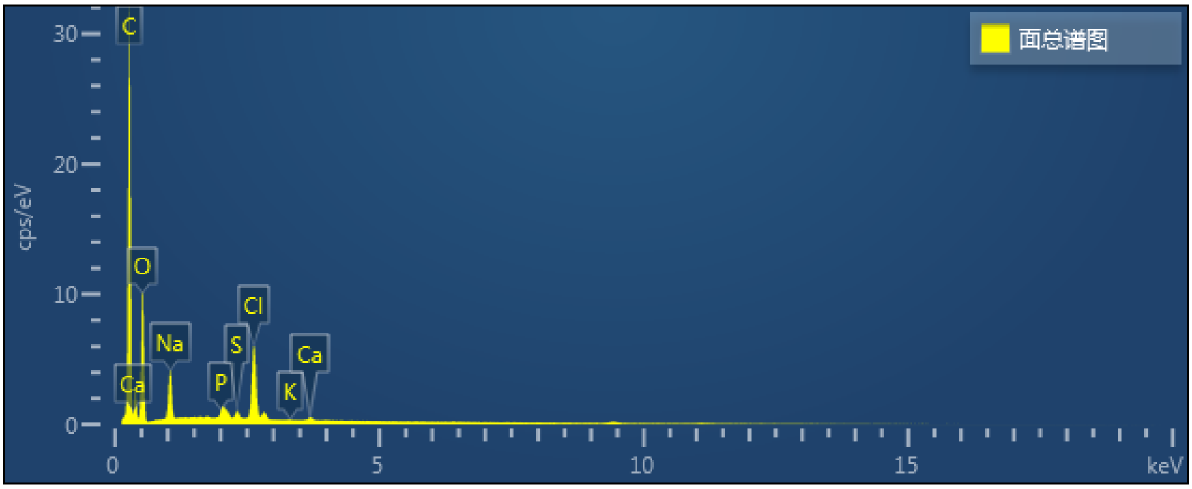

The elemental distribution of scaffolds

Element	Atomic perce of DBM-ECM scaffolds (%)	Element	Atomic perce of DBM-ECM scaffolds (%)	Element	Atomic perce of DBM-ECM scaffolds (%)	Element	Atomic perce of DBM-ECM scaffolds (%)
C	79.33	C	75.07	C	76.76	C	77.77
O	14.2	O	21.36	O	19.61	O	20.4
Na	3.03	Na	0.73	Na	1.95	Na	0.93
P	0.21	P	0.82	P	0.13	P	0.04
S	0	S	0.08	S	0.09	S	0.07
Cl	2.96	Cl	0.52	Cl	1.42	Cl	0.75
K	0.01	K	0	K	0.01	K	0
Ca	0.26	Ca	1.41	Ca	0.02	Ca	0.04
Total:	100	Total:	100	Total:	100	Total:	100

The paired T-tests was used for the analysis for difference for each element between DBM and DBM-ECM groups. The P values for distribution of C, O, Na, P, S, Cl, K and Ca in DBM and DBM-ECM scaffolds were 0.0466, 04584, 0.0218, 0.1744, 0.0447, 0.0496, 0.2070 and 0.2712.
