# Supplementary material for: Macrophage-Mediated Bone Formation in Scaffolds Modified With MSC-Derived Extracellular Matrix Is Dependent on the Migration Inhibitory Factor Signaling Pathway
Source: Front Cell Dev Biol. 2021 Sep 21;9:714011. doi: 10.3389/fcell.2021.714011 (PMC8490662; doi:10.3389/fcell.2021.714011)
Supplement: Supplementary file 2 [file Table_1.docx]

Supplemental Table 1 Gene primers for Real time PCR

| Gene | GenBank ID | Sequence | |
| --- | --- | --- | --- |
| TGFβ3 | NM_001511.4 | Forward | GCAAAGTTGTGAAAACAAGAGC |
|  |  | Reverse | ATCCCAGGTTCCTGTCTTTATG |
| BMP1 | NM_001199.4 | Forward | GGGTCATCCCCTTTGTCATTG |
|  |  | Reverse | GCAAGGTCGATAGGTGAACACA |
| BMP2 | NM_001183647.3 | Forward | GACGTTGGTCAACTCTGTTAAC |
|  |  | Reverse | GTCAAGGTACAGCATCGAGATA |
| BMP6 | NM_001718.6 | Forward | AGCGACACCACAAAGAGTTCA |
|  |  | Reverse | GCTGATGCTCCTGTAAGACTTGA |
| WNT5α | NM_001306129.1 | Forward | AATAGATGCAACGATCAGGACA |
|  |  | Reverse | GCAGGTTTCCTCGATTATCCTT |
| WNT7β | NM_058238.3 | Forward | CACAGAAACTTTCGCAAGTGG |
|  |  | Reverse | GTACTGGCACTCGTTGATGC |
| OSM | NM_000177.5 | Forward | CTCCTGGACCCCTATATACGTA |
|  |  | Reverse | CTTCTCCAAGTCCTCGATGTTC |
| FGF2 | NM_003373.4 | Forward | CATCAAGCTACAACTTCAAGCA |
|  |  | Reverse | CCGTAACACATTTAGAAGCCAG |
| PTHLH | NM_198965.2 | Forward | AAGGTGGAGACGTACAAAGAGC |
|  |  | Reverse | CAGAGCGAGTTCGCCGTTT |
| IL-6 | NM_000600.5 | Forward | CACTGGTCTTTTGGAGTTTGAG |
|  |  | Reverse | GGACTTTTGTACTCATCTGCAC |
| OCN | NM_199173.6 | Forward | GAAGTTTCGCAGACCTGACAT |
|  |  | Reverse | GTATGCACCATTCAACTCCTCG |
| OSX | NM_001173467.3 | Forward | GCGGCAAGGTGTATGGCAAGG |
|  |  | Reverse | GCAGAGCAGGCAGGTGAACTTC |
| OPN | NM_000582.3 | Forward | GAAGTTTCGCAGACCTGACAT |
|  |  | Reverse | GTATGCACCATTCAACTCCTCG |
| GAPDH | NM_001289746.1 | Forward | CCAGCAAGAGCACAAGAGGAAGAG |
|  |  | Reverse | GGTCTACATGGCAACTGTGAGGAG |

Supplemental Figure 1

DBM scaffolds

EDS images

| 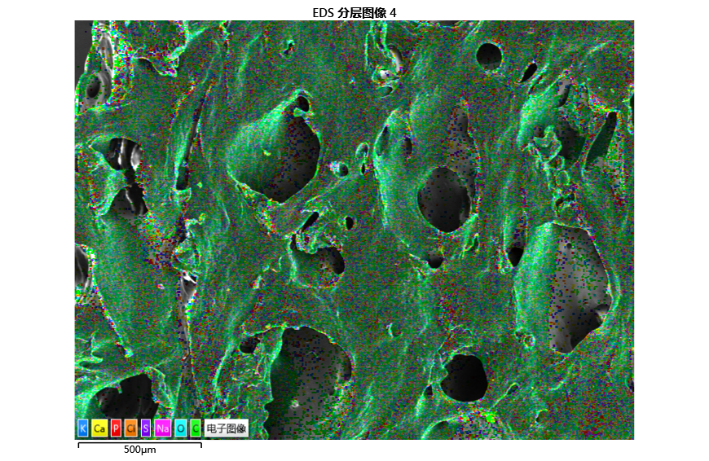 | 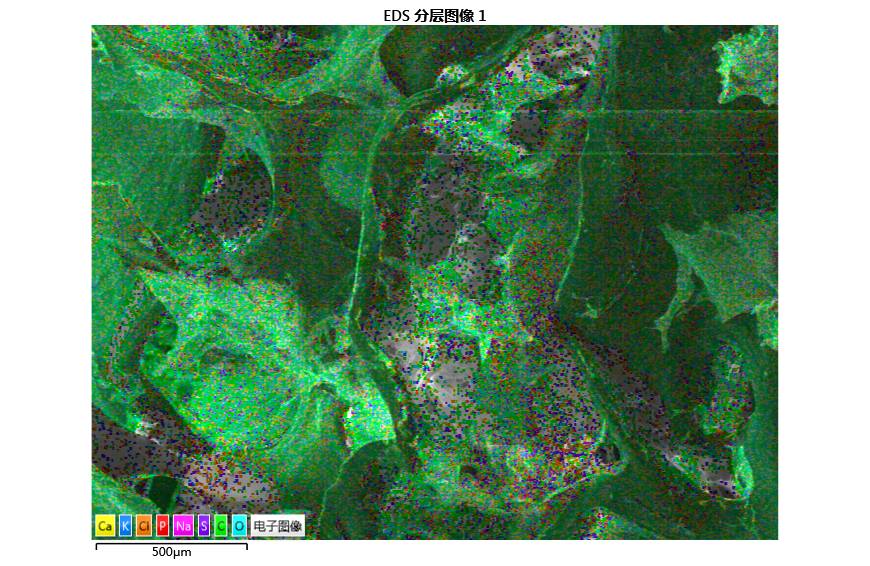 |
| --- | --- |
| 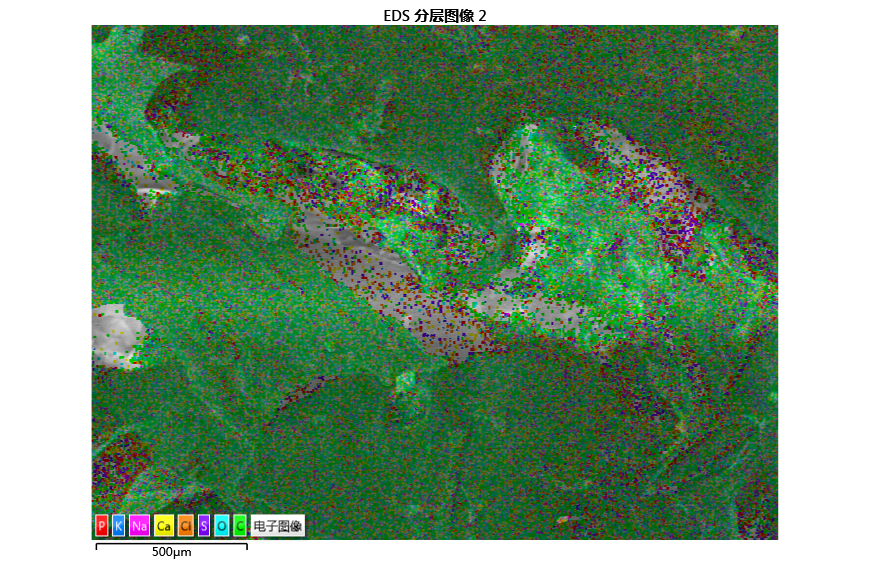 | 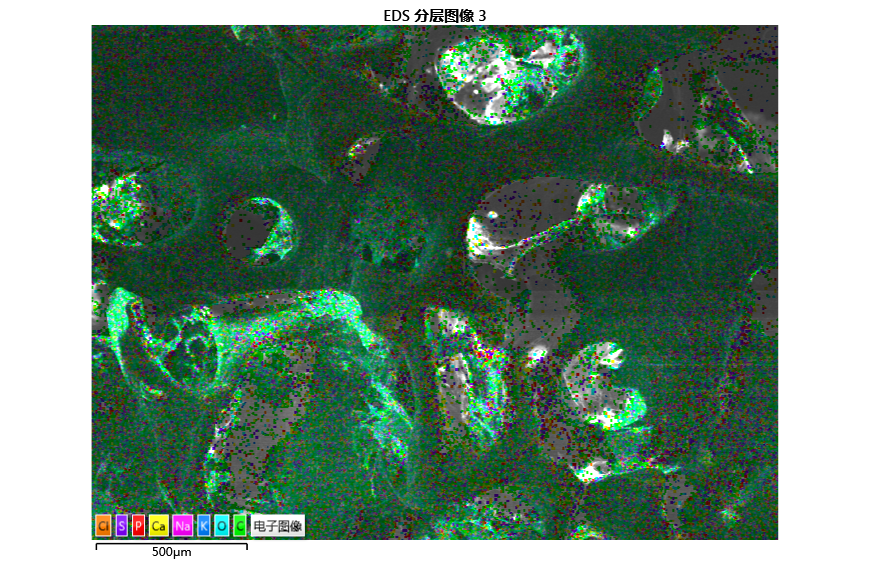 |

| 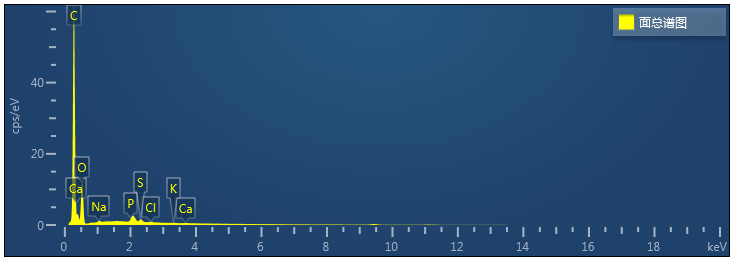 |
| --- |
| 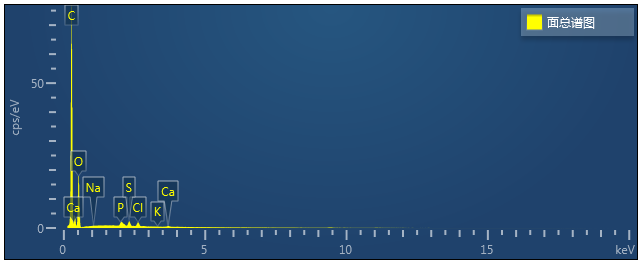 |
| 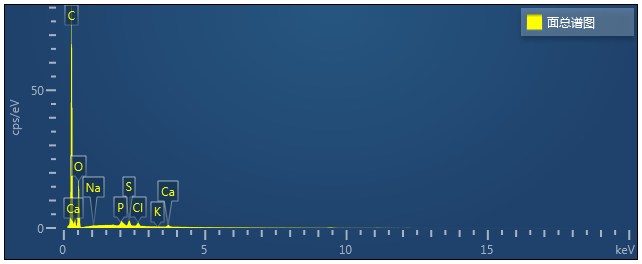 |
| 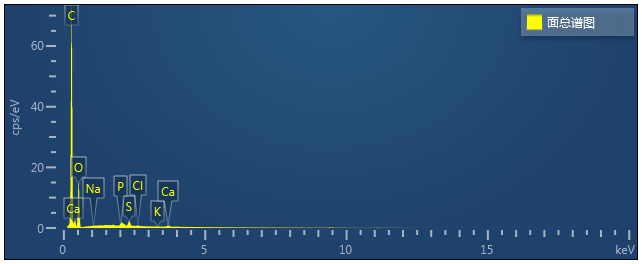 |

The elemental distribution of scaffolds

| Element | Atomic perce of DBM scaffolds (%) | Element | Atomic perce of DBM scaffolds (%) | Element | Atomic perce of DBM scaffolds (%) | Element | Atomic perce of DBM scaffolds (%) |
| --- | --- | --- | --- | --- | --- | --- | --- |
| C | 79.32 | C | 78.92 | C | 79.86 | C | 80.16 |
| O | 20.5 | O | 20.78 | O | 19.8 | O | 19.62 |
| Na | 0.07 | Na | 0.01 | Na | 0.02 | Na | 0.01 |
| P | 0 | P | 0.05 | P | 0.05 | P | 0.02 |
| S | 0.07 | S | 0.12 | S | 0.14 | S | 0.13 |
| Cl | 0.02 | Cl | 0.1 | Cl | 0.09 | Cl | 0.02 |
| K | 0.01 | K | 0 | K | 0 | K | 0 |
| Ca | 0.01 | Ca | 0.02 | Ca | 0.05 | Ca | 0.04 |
| Total: | 100 | Total: | 100 | Total: | 100 | Total: | 100 |

DBM-ECM scaffolds

EDS images

| 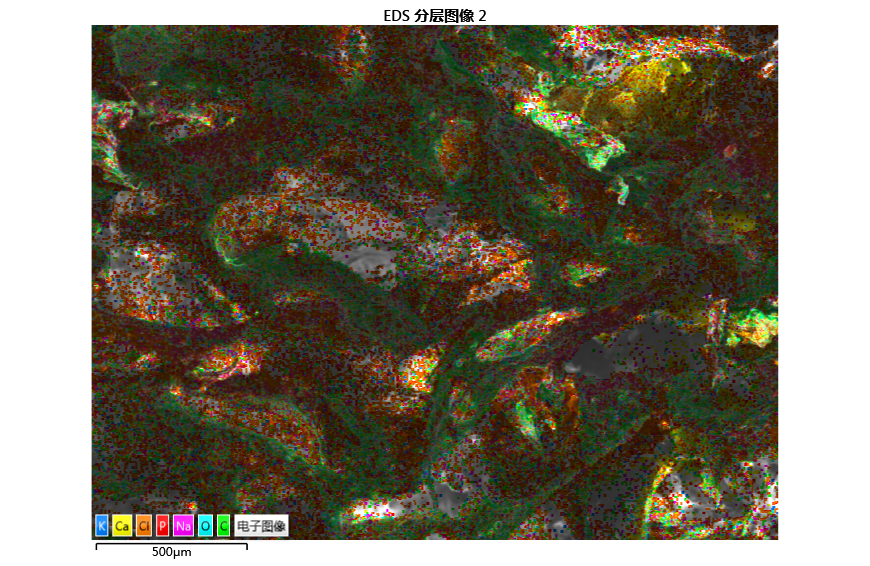 | 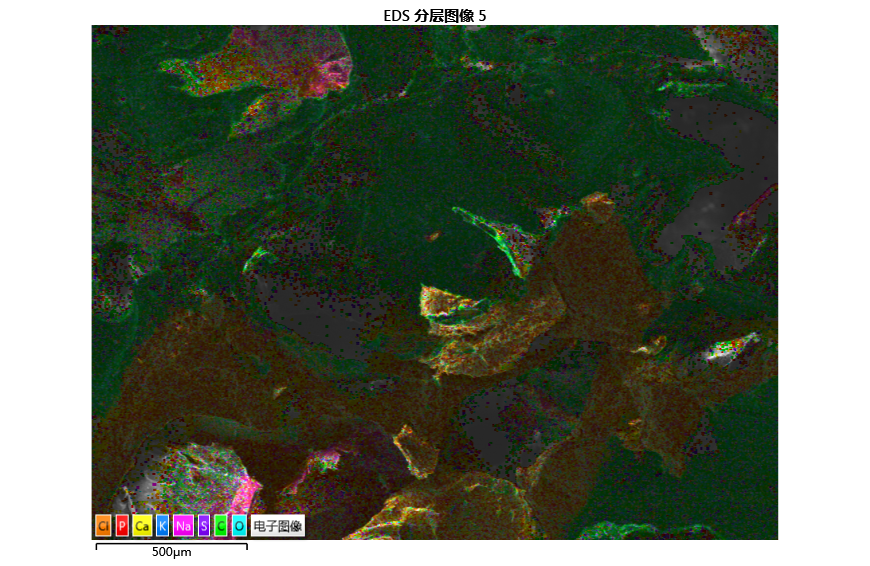 |
| --- | --- |
| 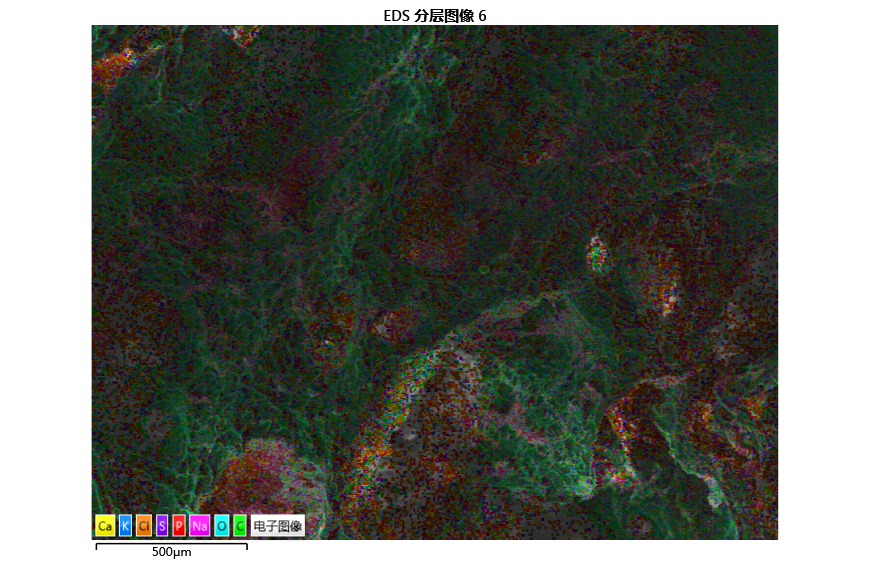 | 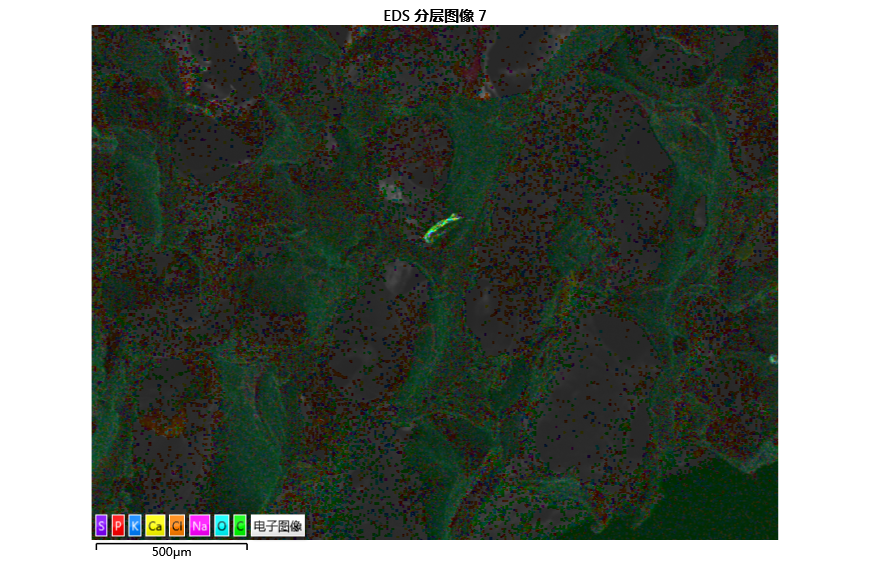 |

| 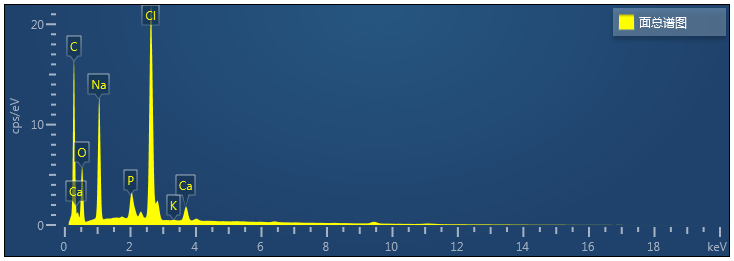 |
| --- |
| 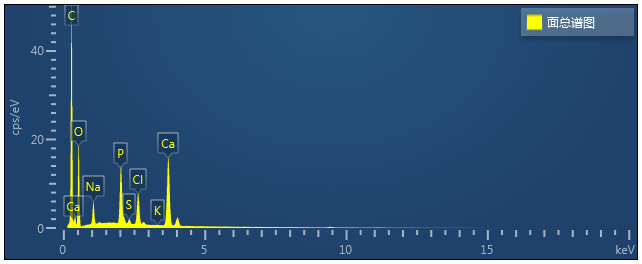 |
| 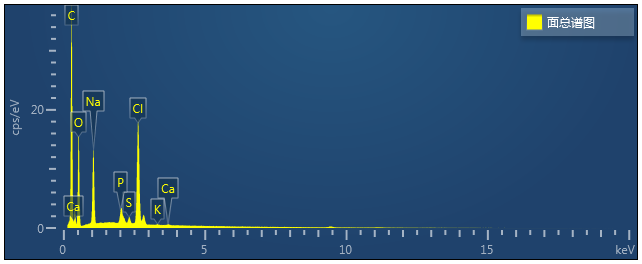 |
| 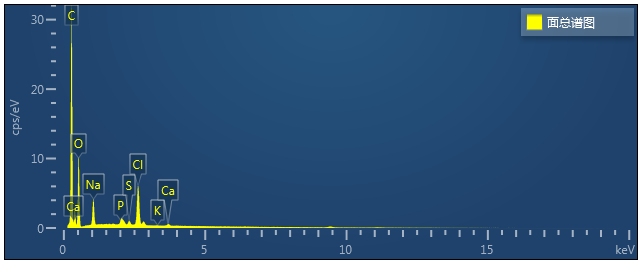 |

The elemental distribution of scaffolds

| Element | Atomic perce of DBM-ECM scaffolds (%) | Element | Atomic perce of DBM-ECM scaffolds (%) | Element | Atomic perce of DBM-ECM scaffolds (%) | Element | Atomic perce of DBM-ECM scaffolds (%) |
| --- | --- | --- | --- | --- | --- | --- | --- |
| C | 79.33 | C | 75.07 | C | 76.76 | C | 77.77 |
| O | 14.2 | O | 21.36 | O | 19.61 | O | 20.4 |
| Na | 3.03 | Na | 0.73 | Na | 1.95 | Na | 0.93 |
| P | 0.21 | P | 0.82 | P | 0.13 | P | 0.04 |
| S | 0 | S | 0.08 | S | 0.09 | S | 0.07 |
| Cl | 2.96 | Cl | 0.52 | Cl | 1.42 | Cl | 0.75 |
| K | 0.01 | K | 0 | K | 0.01 | K | 0 |
| Ca | 0.26 | Ca | 1.41 | Ca | 0.02 | Ca | 0.04 |
| Total: | 100 | Total: | 100 | Total: | 100 | Total: | 100 |

The paired T-tests was used for the analysis for difference for each element between DBM and DBM-ECM groups. The P values for distribution of C, O, Na, P, S, Cl, K and Ca in DBM and DBM-ECM scaffolds were 0.0466, 04584, 0.0218, 0.1744, 0.0447, 0.0496, 0.2070 and 0.2712.
